# Minimally Invasive Image-Guided Transgluteal Approach for Resection of a Sciatic Nerve Tumor: A Technical Note

**DOI:** 10.7759/cureus.37885

**Published:** 2023-04-20

**Authors:** David S Bailey, Lekhaj C Daggubati, Neel Patel, Kimberly Harbaugh, Elias Rizk

**Affiliations:** 1 Neurosurgery, Penn State Health Milton S. Hershey Medical Center, Hershey, USA

**Keywords:** sciatic tumor, transgluteal, minimally invasive, intraoperative ultrasound, extra pelvic leiomyoma

## Abstract

There are a variety of surgical approaches to lesions around the sciatic notch. Historically, peripheral nerve surgeons prefer an infragluteal approach involving a large incision with reflection of the gluteus maximus to better visualize the operative field. This approach was imperative when lesion localization was imprecise. Comparatively, orthopedic surgeons prefer a muscle-splitting, transgluteal approach to operate on the static structures of the posterior hip. The transgluteal approach is significantly less morbid, allowing for same-day discharge and less extensive rehab given preservation of the gluteal muscle.

In this article we describe the use of dynamic ultrasound imaging to localize and aid in the resection of three unique tumors around the sciatic notch using a minimally invasive, tissue-sparing, transgluteal technique. We offer a comprehensive description of the benefits, anatomic considerations, and nuances of using a transgluteal approach for the resection of lesions at the sciatic notch.

## Introduction

Sciatica is commonly caused by spinal pathology, including disc herniation and spinal stenosis. Disc herniation accounts for 85% of cases [1]. Less frequently, sciatica may be attributed to the sciatic nerve's extraspinal entrapment secondary to numerous etiologies, including piriformis syndrome and tumor growth in and around the sciatic notch [2]. This presentation is typically more insidious, with constant pain unaffected by positional change [2]. Recognizing tumor growth as the etiology for lumbosacral radiculopathy may be difficult due to its infrequency, but early recognition is important to improve outcomes and decrease unnecessary medical interventions [2]. These tumors' common pathology includes schwannomas, neurofibromas, and malignant nerve sheath tumors, while other tumor types are much less common [3].

Surgical techniques for tumors around the sciatic notch can be influenced by numerous factors, including tumor invasion patterns. Invasion through the sciatic notch typically requires a combined transabdominal and posterior approach [4-11]. Neoplasms that remain extra-pelvic may be resected using only a posterior approach. There are two main techniques for the posterior approach, infragluteal and transgluteal. The infragluteal approach requires a large question-mark incision and reflection of the gluteus maximus to visualize the sciatic notch's neurovascular structures. Historically, peripheral nerve surgeons have preferred this approach due to the wide visualization of the sciatic nerve and surrounding structures [12]. An alternative is a transgluteal approach. For this, a smaller incision over the gluteus muscle is made, and the fibers of the gluteus maximus are retracted to visualize the sciatic nerve and accompanying structures. This alternative method is the classic approach for posterior access to the hip used by orthopedic surgeons [12]. Comparatively, the transgluteal method offers less postoperative morbidity and a quicker return to normal activities due to the smaller incision and intact attachments of the gluteus maximus [13]. More recently, peripheral nerve surgeons have increasingly adopted this approach. 

This report details three cases using a modified, minimally invasive, image-guided version of the transgluteal approach to resect an extra pelvic mass compressing the sciatic nerve. First, we demonstrate the utility of intra-operative x-ray and ultrasound for precise localization of the neoplasm, enabling the transgluteal approach for peripheral nerve procedures. In addition, we discuss the pros and cons of an infragluteal versus a transgluteal approach to the sciatic notch in peripheral nerve surgery. IRB/ethics committee approval was not required for this report. Due to the anonymous nature of the cases presented, patient consent was not sought.

## Case presentation

Case 1

**Figure 1:**
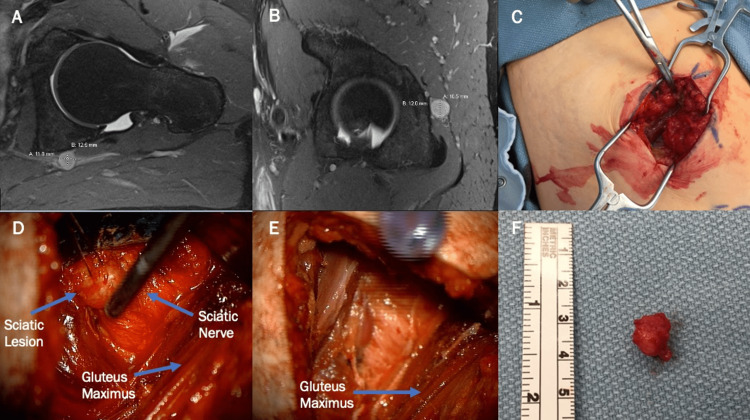
Case 1 preoperative and intraoperative imaging The patient had a 1.1x1.2x1.1cm left sciatic homogenous lesion off the sciatic nerve as it exits the sciatic notch as seen on the axial (A) and sagittal (B) T1 contrast MRI. A minimally invasive linear incision over the lesion (C) was used to use a transgluteal approach to the sciatic notch revealing the lesion (D & E). In addition, an en bloc resection was achieved (F).

This is a 37-year-old male with a prior medical history of right-sided degenerative disk disease with disc protrusion at L5-S1 compressing the right S1 nerve root on MRI. This was successfully managed with non-surgical treatment, including epidural injection, physical therapy, and chiropractic manipulation. He subsequently developed non-specific low-back pain and a lateral, dull, aching pain on his left side. Still, he returned later that month with new left-sided radiculopathy, which worsened while sitting. The patient was referred to an orthopedic spine clinic, where he was scheduled for another epidural injection and a follow-up MRI. Lumbar MRI revealed no significant changes from the prior study, ultimately not explaining the left-sided radiculopathy. In addition, the patient received a sciatic nerve block which worsened his symptoms. Given concerns for a sciatic nerve lesion, a pelvic MRI was ordered and revealed a 1.3cm T1 isointense, T2 hyperintense lesion abutting the sciatic nerve posterior to the left acetabulum (Figure 1). The patient was offered and accepted surgical intervention due to uncontrolled pain.

Following anesthesia, the patient was positioned prone onto the operative table under and connected to neuromonitoring. Using preoperative imaging as guidance, fluoroscopy was then used to localize the tumor. A linear incision was made slightly above where the tumor was located. Dissection was done to visualize the gluteus maximus. The muscle fascia was sharply incised, and the fibers of the gluteus maximus were spread apart with minimal blood loss. The tumor was able to be palpated at a depth of 6cm. The superficial fascia was dissected off the nerve under the microscope, allowing the tumor to be fully visualized and confirmed via intraoperative nerve stimulation. Kitner dissectors were used to dissect the nerve fibers of the tumor. The final pathology revealed that the tumor was a schwannoma. The patient did well positively and was discharged postoperative day one. In a postoperative follow-up, the patient was pain-free from his radicular left-sided pain.

Case 2

**Figure 2:**
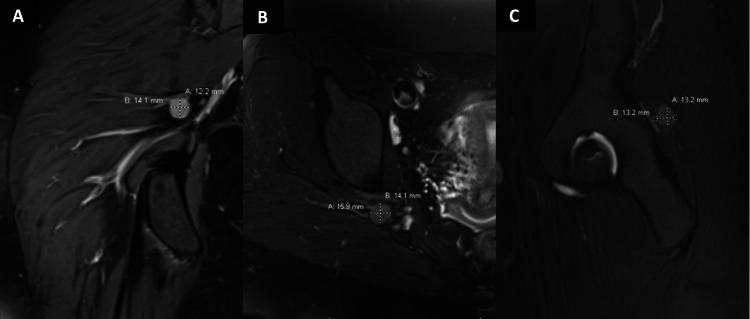
Case 2 preoperative imaging Patient's preoperative MRI of the right leg shows an approximately 1.4cm x1.2cm x 1.5cm contrast-enhancing lesion (A: coronal) that is isointense on T2 fast-saturated imaging (B: axial & C: sagittal).

A 20-year-old female with a prior medical history of non-Hodgkin's lymphoma presented with one year of right-sided hip pain. It is a 10/10 sharp pain that evolved to radiate down to her right lateral ankle for the past three months. A T1 MRI with contrast of the hip revealed a 1x3x1.4cm homogenously hyperintense lesion causing compression of the sciatic nerve (Figure 2). Due to the unrelenting pain and concern for malignancy, the patient requested to proceed with the operation.

**Figure 3:**
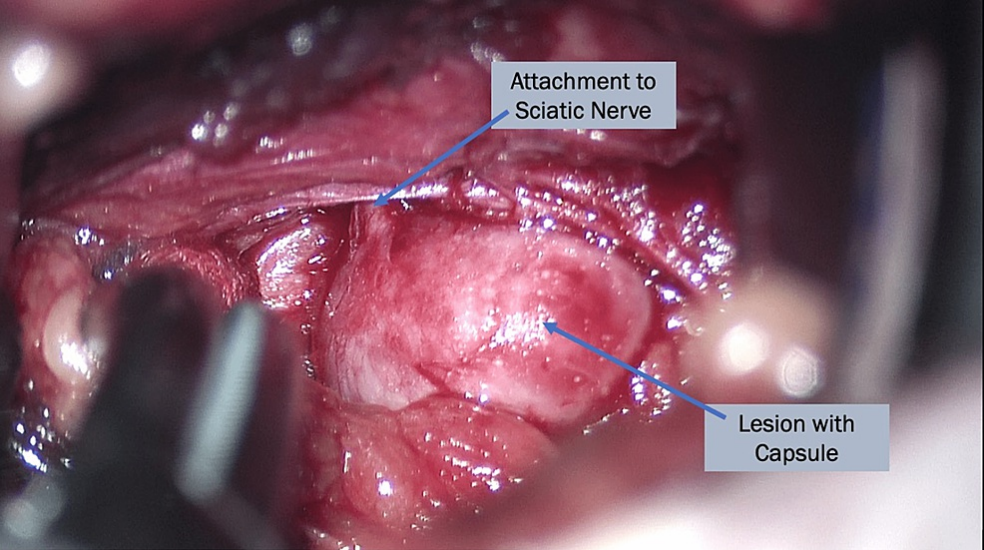
Case 2 intraoperative photograph Intraoperative imaging of Case 2 showing the sciatic notch lesion attached to the sciatic nerve. The lesion was bluntly dissected while ensuring no surrounding vasculature was injured. Palpation for arteries, intraoperative stimulation for functional nerve fibers, and ultrasound for localization were used to achieve the minimally invasive surgery (MIS) approach safely.

After anesthetizing, the patient was positioned prone onto the operative table. Intraoperative ultrasound was attempted prior to the incision to localize the lesion, but the lesion was not easily visualized due to its depth. Intraoperative fluoroscopy was then utilized to approximate the lesion's location by relating the lesion relative to the sciatic notch on the preoperative imaging and intraoperative fluoroscopy. Ultimately, the lesion's approximate location was estimated as being slightly superior to the acetabulum and lateral to the pelvic rim's medial edge. An overlaying 4cm transverse incision in the gluteal region was made. At the level of the gluteus maximum fascia, the hyperechoic lesion could be identified by ultrasound. The fibers of the gluteus maximus were split in line with the fiber directionality in an atraumatic fashion. At this point, the tumor was able to be palpated. It was determined to be slightly superior and lateral to the incision. Self-retaining retractors were utilized. The lesion was intimately associated with the sciatic nerve (Figure 3). Nerve stimulation over the lesion was performed without any movement. At this point, the neoplasm was resected en bloc with preservation of the sciatic nerve. The patient was discharged home the same day after a brief stay in the post-acute care unit. Follow-up in the postoperative period revealed resolution of symptoms, but she still had some residual pain in her buttock region that was no longer associated with radicular symptoms. Pathology for this lesion was determined to be leiomyoma.

Case 3

**Figure 4:**
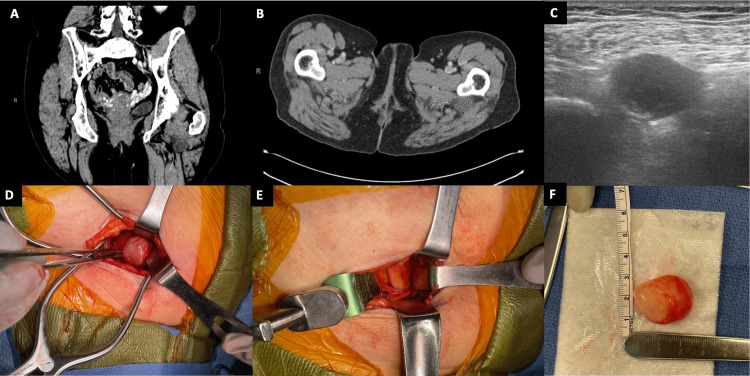
Case 3 preoperative and intraoperative imaging CT-contrasted scan of pelvis with axial (A) and coronal (B) imaging demonstrating an approximately 3x2.4x2.1cm lesion. (C) Intra-op ultrasound of the lesion. Series of intra-op imaging demonstrates the lesion in-situ (D), as well as the sciatic nerve after resection (E), and the lesion post-excision (F).

A 67-year-old retired nurse with a history of breast cancer now on anastrozole presented with right-sided groin pain. CT imaging revealed an incidental mass next to the left sciatic nerve measuring about 3x2.4x2.1cm. No treatment was pursued at that time. At one-year follow-up, the patient was now reporting that she could feel the mass while sitting or lying down and described a buzzing pain in the left sciatic nerve distribution. CT scan of the pelvis with contrast revealed stable size of the lesion (Figure 4). However, since she was now symptomatic the decision was made to move forward with surgical resection.

The patient was brought to the operating room and placed in the prone position. Ultrasound was used to visualize the mass and mark out the borders. The sciatic nerve was also mapped. Linear incision was completed directly over the lesion. Dissection was completed through subcutaneous fat to the gluteal muscle fascia. Gluteal muscles were then separated with blunt dissection using Kitners, finger dissection, and retraction. The mass was stimulated with no movement in the sciatic nerve distribution. The mass was circumscribed and removed en bloc. The procedure went without complication and the patient was discharged same day. Postoperatively the patient had resolution of her presenting symptoms. Pathology came back as myxoma.

## Discussion

Lumbar radiculopathy is most commonly caused by disc herniation or other spinal pathology. Less commonly, sciatica may be caused by distal lesions of the sciatic nerve, including piriformis syndrome and tumor compression. Identifying this less common etiology requires a high degree of clinical suspicion to avoid delay in diagnosis which can prolong patient suffering, and increase healthcare expenditure. An analysis by Bickel et al. demonstrated that the average time to diagnose patients with tumor compression causing sciatica was 11.9 months. The 32 patients studied typically presented with an insidious, constant, progressive pain unresponsive to positioning. In addition, half of those studied could point to a specific point along the extra-spinal course of sciatic pain. The authors concluded that the pelvis routine anteroposterior plain radiography might be helpful as a part of the initial workup for lumbar radiculopathy [2]. The difficulty in diagnosing this rare pathology was demonstrated in case 1. This patient had a known history of spinal pathology and contralateral sciatica attributed to radicular symptoms, despite a lack of anatomic correlation on MRI. This ultimately led to a seven-month interval between symptom onset and diagnosis. Once recognized, the treatment is surgical resection, which can be done with a minimally invasive technique.

The primary goal of minimally invasive surgery is to cause as little disruption to tissue as possible. Ultimately, this leads to less blood loss, shorter hospital stays, less tissue injury, and improved recovery. Additionally, the use of smaller incisions provides an improved cosmetic outcome. Minimally invasive surgery has been utilized in neurosurgery for some time [14,15]. This includes spine surgery as well as intracranially for tumor resection and aneurysm clipping [16-18]. However, in peripheral nerve surgery, there has been less innovation toward a minimally invasive technique; many surgical methods remain like those described in the 1920s [13].

The classic approaches to the gluteal region include an infragluteal approach versus a transgluteal approach. The transgluteal approach was first described at the turn of the 20th century [19,20]. Shortly thereafter, the infragluteal approach was described by Stookey [21]. Henry further described the infragluteal approach and warned against the transgluteal technique due to the feared disruption of vascular planes [22]. A major concern of the transgluteal approach was causing damage to the gluteal region veins, which may not be recognized with this technique [22]. Injury to one or more branches of the inferior gluteal is also a possibility and may lead to atrophy or dimpling of the gluteus muscle, postoperatively.

While some neurosurgeons still favored the transgluteal approach, the infragluteal approach became the primary technique used by peripheral nerve surgeons, who preferred the wider visualization offered by the reflection of the gluteus maximum [12]. This improved visualization was paramount when the localization of lesions and their extent was imprecise, limited to that which could be revealed by physical exam [12]. This is a limitation orthopedic surgeons do not face, operating on a fixed structure such as the hip and gravitating to the transgluteal approach.

Innovations in imaging have allowed the peripheral nerve surgeon to no longer rely exclusively on the physical exam's inaccurate estimates. Now, neurosurgeons can preoperatively visualize the position and size of the lesion in question and plan accordingly. Furthermore, cadaver research by Socolovsky et al. demonstrates that differences in visualization between the infragluteal and transgluteal approaches may not be so significant [13]. They report that with a transgluteal approach, they can visualize a mean of 11.5cm of the surface beneath the gluteus maximus. In addition, they could visualize the entirety of the sciatic notch though they were limited in their view distally. To overcome this, they describe a transverse incision as opposed to the classic vertical used in transgluteal approaches. The benefits of this include an improved cosmetic outcome. In addition, if intraoperatively extension is needed distally, a transverse incision may easily be converted into the question mark incision required for an infragluteal approach [13].

Our minimally invasive, image-guided transgluteal approach utilizes advances in intraoperative imaging to minimize the incision and tissue disruption. We demonstrate the use of intraoperative ultrasound and fluoroscopy to plan a precise location for our incision. While ultrasound was ineffective prior to incision due to the lesion's depth in case 2, fluoroscopy allowed for a precise target in both cases. We created a 4-5cm transverse incision and used intraoperative ultrasound to reorient towards the lesion within the small incision. This incision is significantly smaller than the large question mark incision used in the infragluteal approach and smaller than commonly used in the transgluteal approach. However, this smaller window still allowed us adequate target visualization, complete resection, and a successful outcome.

In addition to the open approaches discussed, an endoscopic approach to the gluteal region is commonly used for the treatment of deep gluteal syndrome (DGS) which is a broader term to describe the many causes of non-discogenic sciatica that was previously encompassed under “piriformis syndrome” [23]. Endoscopic surgery for the treatment of DGS has extremely good outcomes with low morbidity [24,25]. While this technique has proven successful in orthopedics and may be less invasive than a transgluteal approach, it may not be appropriate for the resection of gluteal region tumors. Endoscopic surgery requires advanced training and routine utilization to reduce surgical morbidity. Tumors in this region are rare, and peripheral nerve surgeons lack the volume to appropriately hone their endoscopic technique. Thus, the more intuitive transgluteal approach would be expected to have better outcomes. In addition, the endoscopic technique requires supine positioning with significant contralateral table tilt [25]. This positioning precludes conversion to an open procedure if necessary. For these reasons we would argue that a minimally invasive, transgluteal approach is a better option than endoscopic surgery for resection of gluteal region tumors.

Finally, while tumor pathology in this region is commonly schwannomas, as described in case 1, neurofibromas, or malignant nerve sheath tumors, the pathology demonstrated in the second case was a deep soft tissue leiomyoma. This is an especially unusual finding as leiomyoma is typically found in the uterus [26]. Deep soft tissue leiomyoma, though rare, may occur in somatic soft tissue, retroperitoneum, and the abdominal cavity [27]. The treatment of these lesions requires wide local excision. In addition to the patient's history of lymphoma, this tumor pathology required further screening for additional lesions. The third case describes a myxoma which is another unusual lesion for this region but still more common than a leiomyoma. Myxomas are benign tumors of the musculoskeletal system that are commonly painless, but can cause stretch or compression of nearby structures, in our instance the sciatic nerve, causing symptoms. Surgical excision is curative [28].

## Conclusions

The constant march towards minimally invasive surgery is important to improve patient outcomes. Here we demonstrated a minimally invasive image-guided technique for peripheral nerve lesions at the sciatic notch. Intraoperative imaging, including ultrasound and fluoroscopy, allowed for specific lesion localization and a tailored incision. This improved precision improves minimally invasive peripheral nerve surgery techniques and further negates the need for the more invasive infragluteal approach.
